# Complete genome sequence of *Xanthomonas arboricola* pv. *pruni* strain xap_25-1136 isolated from cherry laurel in Pennsylvania, USA

**DOI:** 10.1128/mra.00352-26

**Published:** 2026-06-04

**Authors:** Sara R. May, Luis Fernando Flores-Lopez, Lauren E. Carneal, Joshua J. Giesler, Richard S. Gonzalez Aquino, Damiana S. Rojas, Sharifa G. Crandall, Veronica Roman-Reyna

**Affiliations:** 1 Department of Plant Pathology and Environmental Microbiology, The Pennsylvania State University 171650, University Park, Pennsylvania, USA; 2 One Health Microbiome Center, Huck Institutes of the Life Sciences, The Pennsylvania State University 124474, University Park, Pennsylvania, USA; University of Manitoba, Winnipeg, Manitoba, Canada

**Keywords:** *Xanthomonas arboricola pv. pruni*, cherry laurel, whole genome

## Abstract

We report the complete genome sequence of the bacterium *Xanthomonas arboricola* pv. *pruni*, isolated from symptomatic cherry laurel leaf tissue submitted to the Pennsylvania State University Plant Disease Clinic by a commercial ornamental plant nursery. Genome size is 5,271,940 bp and consists of one chromosome and four plasmids.

## ANNOUNCEMENT

**Fig 1:**
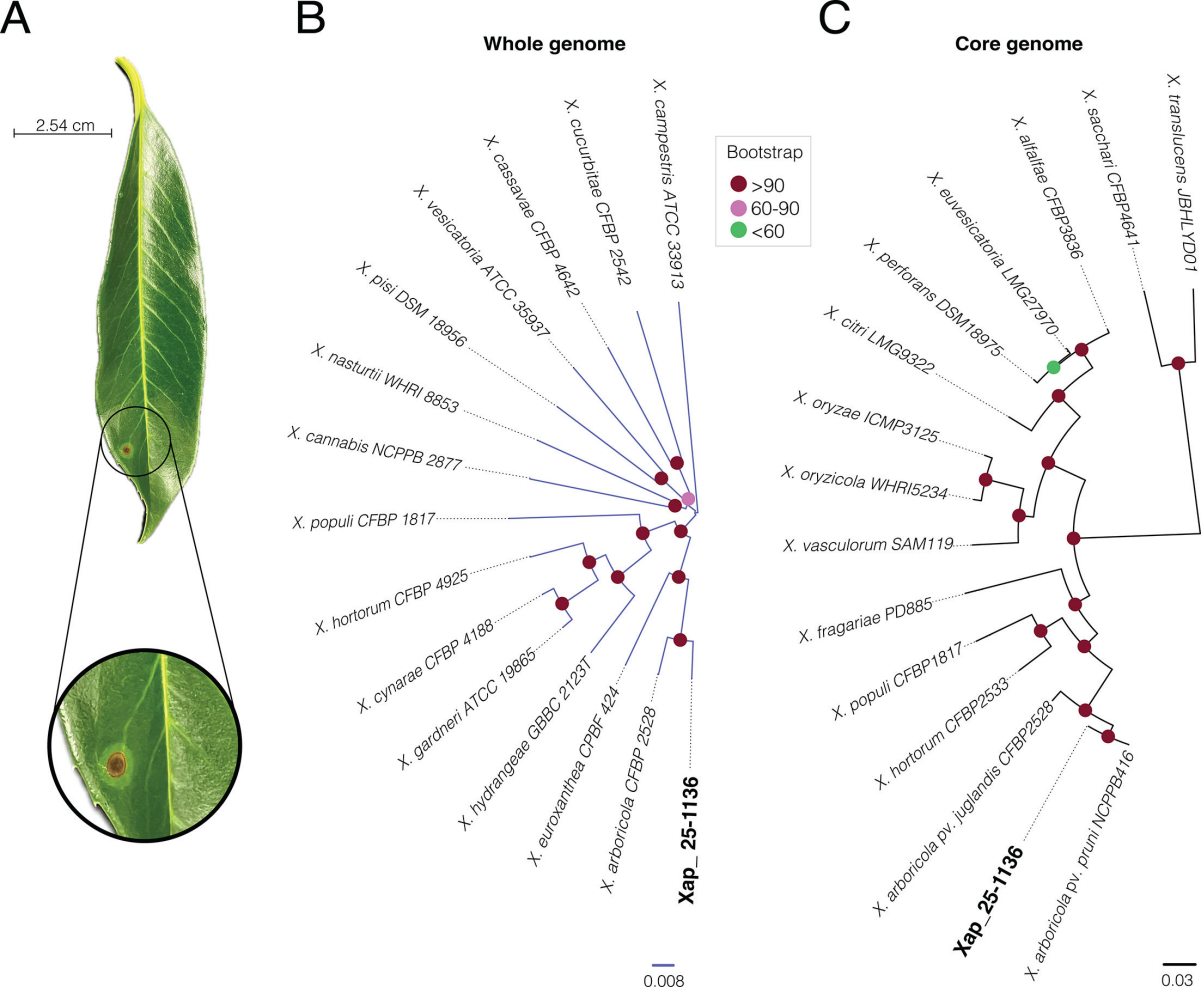
Isolation and identification of *Xanthomonas arboricola* pv. *pruni* from symptomatic leaf tissue. (**A**) Symptomatic cherry laurel leaf showing a necrotic lesion and chlorotic halo. (**B**) Whole-genome phylogenomic tree generated with TYGS server and default parameters; branches are shown in blue. (**C**) Maximum-likelihood phylogenetic tree inferred from a core-gene alignment using the MFP model and 1,000 bootstrap replicates in IQ-TREE (v3.0.0). For both trees, circles on nodes indicate bootstrap support values, and circle color represents support ranges. Leaf photo: S.G. Crandall.

*Xanthomonas arboricola* pv. *pruni* (Xap) is an economically important bacterial plant pathogen that causes bacterial spot on *Prunus* species, including peach, apricot, and plum, as well as ornamental hosts such as cherry laurel (*Prunus laurocerasus* ‘Schipkaensis’ L.) (1, 2). Although complete Xap genome sequences from stone fruits are available, no genomes from ornamental *Prunus* species have been deposited in GenBank. Here, we report the genome sequence of strain Xap_25-1136, isolated from a symptomatic cherry laurel leaf submitted to the Pennsylvania State University Plant Disease Clinic by a commercial nursery in Lancaster County, Pennsylvania (Fig. 1A).

Symptomatic leaf tissue was surface sterilized and plated onto nutrient agar (NA) to isolate bacterial colonies. After three successive passages to obtain pure cultures, plates were incubated at 28°C for 2 days, and colonies were selected for bioassays and colony PCR. Biochemical characterization of Xap_25-1136 indicated positive reactions for esculin and gelatin hydrolysis, citrate utilization, and levan production, and negative reactions for oxidase and starch hydrolysis (3). For molecular identification, direct colony PCR targeting the 16S rRNA gene was performed using universal primers 27F/1492R (5′-AGAGTTTGATCMTGGCTCAG-3′/5′-TACCYTACCTTGTTACGACTT-3′) (4), and the amplicon was Sanger sequenced at the Huck Institutes of the Life Sciences, Genomics Core Facility at the Pennsylvania State University. BLASTn analysis on the 16S rRNA sequence showed 100% coverage and identity to *X. arboricola* (accession no. PZ213069) (5).

For whole-genome sequencing, a colony was plated on NA and incubated at 28°C for 2 days, and high-molecular-weight DNA was extracted using the Promega HMW DNA Extraction Kit according to the manufacturer’s instructions (Madison, WI). DNA was not sheared, and fragments shorter than 600 bp were removed using the AMPure protocol following the manufacturer’s website protocol (Indianapolis, IN). Sequencing libraries were prepared with the Oxford Nanopore Rapid Barcoding Kit SQK-RBK114.24 and run on an Oxford Nanopore MinION platform with a R10.4 flow cell (Oxfordshire, UK). Default parameters were used for all software except where noted. Live base calling was performed using MinKNOW (v25.09.16) and Dorado (v1.4.0) (6), and the genome was assembled using Flye (v2.9.6) with nano-hq model (7). The assembly was 5,271,940 bp, with genome coverage ranging from 4 to 2,000×, and the assembly completeness was estimated using BUSCO (v6.0.0) (8) (Table 1). The assembly consisted of one chromosome and four plasmids (Table 1). Genome annotation was performed with Bakta, which predicted 4,515 genes (9). Taxonomic analysis using the TYGS server confirmed that Xap_25-1136 belongs to *X. arboricola* (10) (Fig. 1B).

**TABLE 1:** Genomic data for strain Xap_25-1136

Feature	Xap_25-1136
ONT raw read number	2,293,065
Genome size N50	5,271,940 bp; 5,063,698 bp
CDS (total)	4,515
%GC	65.1%
BUSCO results using Xanthomonadales_odb12 lineage	Complete: 99.4; fragmented: 0.3%; missing: 0.3%.
Plasmids name; size; NCBI BLASTn with >80% similarity and >60% coverage	25-1136_p89; 89,111 bp; *Xanthomonas* sp. LMG31884_p73
	25-1136_p88; 88,881 bp; *X. hortorum* pv. *pelargonii* pOSU498
	25-1136_p15; 15,497 bp; *Acinetobacter radioresistens* plasmid p4-E3263934
	25-1136_p14; 14,753 bp; *A. gandensis* M1_NDM_tet(X3) plasmid unnamed4

Core genes were extracted with panaroo (v1.5.2) (11), aligned with mafft, and used to infer a maximum-likelihood phylogeny with IQ-TREE (v3.0.0) (12), which supported the assignment of Xap_25-1136 to *X. arboricola* pv. *pruni* (Fig. 1C). The Xap_25-1136 genome encodes heavy metals resistance genes (*copABRF*, *pcoABCDF*, *czrAB*, *cusAB*) and the gene tetR (13). The type III effector gene *xopE3*, specific to Xap, was identified, and no transcription activator-like effectors were predicted (5, 14, 15). This work represents a collaborative effort between the Pennsylvania State University Plant Disease Clinic and a graduate student course in the Department of Plant Pathology and Environmental Microbiology in 2025.

## Data Availability

The 16S rRNA sequence (accession no. PZ213069) and the whole unrotated genome sequence (JBWJFR000000000.1) are deposited in GenBank under BioProject PRJNA1439916 and BioSample SAMN56597282. The SRA accession number is SRR37956053. The biochemical assays photos are deposited at 10.6084/m9.figshare.31879537.
